# Tinigible Body Macrophages in Regulation of Germinal Center Reactions

**DOI:** 10.1155/1998/38923

**Published:** 1998

**Authors:** John P. Smith, Gregory F. Burton, John G. Tew, Andras K. Szakal

**Affiliations:** 1 Department of Anatomy Division of Immunobiology Virginia Commonwealth University Medical College of Virginia Richmond Virginia USA; 2 Department of Microbiology and Immunology Virginia Commonwealth University Medical College of Virginia Richmond Virginia USA; 3 Department of Anatomy and Cell Biology ECU School of Medicine Brody Building, 7N-94 Greenville NC 27858 USA

**Keywords:** Antigen presentation, apoptosis, iccosomes, macrophage, microenvironment, prostaglandin

## Abstract

Tingible body macrophages (TBM), long thought simply as scavengers of apoptotic lymphocytes, are located in the unique microenvironment of germinal centers in close proximity to antigen-retaining follicular dendritic cells (FDC). Observations that TBM endocytose FDC-iccosomal (immune-complex coated bodies) antigen suggested that TBM might present this antigen and help regulate the germinal center reaction. To test for antigen presentation, the ovalbumin (OVA)-specific T_H_ hybridoma, 3DO-54.8, which produces IL-2 on receiving effective presentation of OVA, were used as responders to OVA-bearing TBM. Results showed that OVA-bearing TBM failed to induce IL-2 production. Furthermore, addition of TBM to IL- 2-inducing positive controls (B cells) not only failed to augment IL-2 production, but rather TBM significantly (55-90%) reduced B-cell induction of IL-2. We found that TBM were rich in prostaglandin by comparison with other nongerminal center lymph node macrophages and that addition of indomethacin to the cultures reversed the inhibitory effect of TBM. Depletion of TBM from enriched preparations, prior to addition to positive control cultures, also abrogated the inhibitory effect on IL-2 production. These data support the concept that TBM, within the unique microenvironment of germinal centers, may be specialized to downregulate the germinal center reaction.


*Developmental Immunology*, 1998, Vol. 6, pp. 285–294
Reprints available directly from the publisher
Photocopying permitted by license only

© 1998 OPA (Overseas Publishers Association)
N.V. Published by license under the
Harwood Academic Publishers imprint,
part of the Gordon and Breach Publishing Group
Printed in Malaysia

### Tingible Body Macrophages in Regulation of Germinal Center Reactions

JOHN P. SMITH[a], GREGORY F. BURTON[b], JOHN G. TEW[b] and ANDRAS K. SZAKAL[a*]

[a]*Department of Anatomy, Division of Immunobiology,;* [b]*Department of Microbiology and Immunology, Virginia Commonwealth University, Medical College of Virginia, Richmond, Virginia*

*(Received 2 November 1996; in final form 3 June 1997)*

Tingible body macrophages (TBM), long thought simply as scavengers of apoptotic lymphocytes, are located in the unique microenvironment of germinal centers in close proximity to antigen-retaining follicular dendritic cells (FDC). Observations that TBM endocytose FDC-iccosomal (immune-complex coated bodies) antigen suggested that TBM might present this antigen and help regulate the germinal center reaction. To test for antigen presentation, the ovalbumin (OVA)-specific $T_H$ hybridoma, 3DO-54.8, which produces IL-2 on receiving effective presentation of OVA, were used as responders to OVA-bearing TBM. Results showed that OVA-bearing TBM failed to induce IL-2 production. Furthermore, addition of TBM to IL-2-inducing positive controls (B cells) not only failed to augment IL-2 production, but rather TBM significantly (55-90%) reduced B-cell induction of IL-2. We found that TBM were rich in prostaglandin by comparison with other nongerminal center lymph node macrophages and that addition of indomethacin to the cultures reversed the inhibitory effect of TBM. Depletion of TBM from enriched preparations, prior to addition to positive control cultures, also abrogated the inhibitory effect on IL-2 production. These data support the concept that TBM, within the unique microenvironment of germinal centers, may be specialized to downregulate the germinal center reaction.

*Keywords:* Antigen presentation, apoptosis, iccosomes, macrophage, microenvironment, prostaglandin

### INTRODUCTION

Macrophages represent a heterogenous population, and macrophage functions are influenced by the microenvironment in which they reside. By definition, tingible body macrophages (TBM), a subset of the mononuclear phagocytes, are unique, large phagocytic cells that reside in germinal centers of secondary lymphoid tissues (Flemming, 1885). TBM contain many phagocytized, apoptotic cells (referred to as tingible bodies) (Flemming, 1885) in various states of degradation (Swartzendruber and Congdon, 1963). Unique to this subset of macrophages is the germinal center microenvironment, characterized functionally by long-term antigen retention on follicular dendritic cells, antigen presentation by B cells to T-helper cells,

*Corresponding author.
Present address: Department of Anatomy and Cell Biology, ECU School of Medicine, Brody Building, 7N-94, Greenville, NC 27858.

TABLE I Antigen Presentation of *In Vivo* Obtained and Exogenous Antigen by TBM-Enriched Cells

| Experiment no. | TBM[a] | TBM + OVA[b] | IL-2 control[c] |
|---|---|---|---|
| 1 | 1,240 ± 165[d] | 25,000 ± 1,800 | 322,000 ± 5,900 |
| 2 | 190 ± 50 | 250 ± 10 | 181,000 ± 1,400 |
| 3 | 300 ± 90 | 950 ± 120 | 364,000 ± 10,240 |
| 4 | 770 ± 100 | 4,340 ± 40 | 60,000 ± 3,600 |

[a] $1.0 \times 10^5$ TBM-enriched cells were cultured for 24 hr with $3 \times 10^4$ 3DO-54.8 $T_{H1}$ cells. Results shown were always greater than control cultures of 3DO cells or TBM alone.

[b] $1.0 \times 10^5$ TBM-enriched cells and 50 $\mu$g OVA were cultured for 24 hr with $3 \times 10^4$ 3DO-54.8 $T_{H1}$ cells.

[c] $3 \times 10^4$ CTL cells were cultured with either 50 $\mu$l IL-2 containing Con A supernatant or 50 $\mu$M human rIL-2.

[d] Data represent $^3$H-thymidine incorporation by $3 \times 10^4$ IL-2-dependent CTLL cells and are expressed as mean cpm ± standard errors of triplicate cultures.

a high rate of somatic mutations, affinity maturation, induction of antibody-forming cells and memory B-cell development (Coico et al., 1983; Szakal et al., 1989; Tew et al., 1990). Although the scavenging function of TBM in germinal centers is obvious, it has also been suggested that TBM may be important in initiating the germinal center reaction (Kamperdijk et al., 1978, 1982).

Regarding the germinal center reaction, it has been shown that germinal center B cells are extremely effective antigen-presenting cells. Three to eight days after a booster immunization, germinal center B cells obtain antigen from FDCs in the form of *i*mmune *c*omplex *co*ated bodiès (iccosomes) and are capable of processing and presenting the iccosomal antigen to T cells in an MHC-restricted manner (Kosco et al., 1988; Szakal et al., 1988). Typically, macrophages phagocytose antigens, process them, express them on class II molecules, and present the processed antigen to T-helper cells. Observations that TBM, like germinal center B cells, also endocytosed iccosomes (Szakal et al., 1988) and that many TBM are class II positive (Smith et al., 1988), suggest that TBM might also be able to present the FDC-derived antigen to T cells (Smith et al., 1988).

The objectives of the present study was to determine if TBM can indeed present antigen and if antigen presentation by TBM may be implicated in the regulation of the germinal center reaction. The results showed that TBM-enriched preparations were not good antigen presenters but were highly effective inhibitors of T-cell lymphokine secretion induced by B cells. Selective depletion of TBM from the enriched preparations and/or addition of the prostaglandin synthesis inhibitor indomethacin to the cultures restored lymphokine activity. Histochemical observations confirmed that TBM are a rich source of prostaglandins. Thus, these data are consistent with the concept that the microenvironment of germinal centers favors an inhibitory role for TBM in the regulation of the germinal center reaction via a prostaglandin-mediated mechanism.

### RESULTS

### Antigen Presentation

Germinal center B cells obtain antigen from FDC for processing and presentation to T cells during a period between 3 and 8 days after booster immunization. Typically, numerous germinal center B cells (20%) were present in the TBM preparation and both TBM and germinal center B cells take up iccosomes (Kosco et al., 1988; Szakal et al., 1988). Therefore, we reasoned that the addition of TBM-enriched cells from an OVA-immune animal to the OVA-specific T-cell hybridoma 3DO-54.8 should result in antigen presentation and IL-2 production. As shown in Table I, there was no evidence of presentation of *in vivo* obtained antigen by the TBM preparation. When antigen was added *in vitro* (TBM + OVA), to be certain that antigen was not limiting, a significant ($p < 0.01$) but very modest level of IL-2 production was detected in two experiments (Table I, Experiments 1 and 4). In both cases, the production of IL-2 was less

TABLE II Effect of TBM-Enriched Cell Preparations on Antigen Presentation by B Cells

| Experiment no. | B cells + OVA[a] | B cells + OVA + TBM[b] | % Suppression | IL-2 control[c] |
|---|---|---|---|---|
| 1 | ⊛165,000[d] ± 9,300 | 16,000 ± 600 | 90.0 | 322,000 |
| 2 | ⊛8,800 ± 330 | 2,700 ± 300 | 70.0 | 22,000 |
| 3 | ✔132,000 ± 8,300 | 13,550 ± 1,600 | 90.0 | 102,000 |
| 4 | ✔96,400 ± 5,700 | 42,700 ± 6,350 | 55.0 | 60,000 |

3DO-54.8 $T_H$ cells ($3 \times 10^4$) were cultured for 24 hr with:
[a]Germinal center B cells ⊛ ($10^6$) or TA3 B-cell hybridoma✔ ($10^4$) and 50 μg OVA (positive control cultures).
[b]Footnote a and $1 \times 10^5$ TBM-enriched cells.
[c]$3 \times 10^4$ CTLL cells were cultured with either 50 μl IL-2 containing Con A supernatant or 50 μM human rIL-2.
[d]Data represent $^3$H-thymidine incorporation by $3 \times 10^4$ IL-2-dependent CTLL cells and are expressed as mean cpm ± standard errors of triplicate cultures. In Experiments 1 to 3, the values for 3DO cells or TBM cultured alone were always less than 500. In Experiment 4, the values for cultures of 3DO alone and TBM alone were 1532 ± 67 and 1087 ± 53, respectively.

TABLE III Indomethacin Relieves Suppression Associated with TBM-Enriched Cells

| Exp. no. | B cells + OVA[a] (positive control) | B cells + OVA + TBM[b] | % of positive control cultures | B cells + OVA TBM + INDO[c] | % of positive control cultures |
|---|---|---|---|---|---|
| 1 | ⊛7,100 ± 700 | 2,600 ± 750 | 37 | 9,076 ± 650 | 129 |
| 2 | ⊛8,800 ± 300 | 2,700 ± 320 | 30 | 7,260 ± 200 | 82 |
| 3 | ✔132,000 ± 8,000 | 13,500 ± 1,600 | 10 | 81,000 ± 1,000 | 61 |
| 4 | ✔96,400 ± 5,700 | 42,700 ± 6,350 | 40 | 66,300 ± 10,300 | 69 |

3DO-54.8 $T_{H1}$ cells ($3 \times 10^4$) were cultured for 24 hr with:
[a]Germinal center B cells⊛ ($10^6$) or TA3 B-cell hybridoma✔ ($10^4$) and 50 μg OVA.
[b]Footnote a and $1 \times 10^5$ TBM-enriched cells. Values for 3DO cells and TBM alone were always less than 500 cpm.
[c]Footnote b and 100 μM indomethacin.
[d]Data represent $^3$H-thymidine incorporation by $3 \times 10^4$ IL-2-dependent CTLL cells and are expressed as mean cpm ± standard errors of triplicate cultures.

than 10% of the response attained in the control when a near optimal level of IL-2 was added.

To determine if TBM might suppress antigen presentation, TBM-enriched cells were cocultured with 3DO cells and B cells in the presence of OVA. The results from four experiments are shown in Table II. A 55-90% suppression of the IL-2 response was observed when TBM-enriched populations were added.

### Effect of Indomethacin on TBM Suppression of Antigen Presentation

Macrophages are a major source of prostaglandins and generally the larger most phagocytically active macrophages are the most prolific prostaglandin producers (Lee and Berry, 1977; Lee and Wong, 1982; Guidos et al., 1987). The striking phagocytic activity of TBM and their large size prompted us to reason that TBM might suppress through a prostaglandin-dependent mechanism. To begin testing this, the prostaglandin synthesis inhibitor indomethacin was added to cultures containing TBM-enriched cells, B cells, and 3DO cells in the presence of OVA. As shown in Table III, the addition of 100 μM indomethacin relieved most of the suppression of IL-2 production in TBM-containing cultures. Controls showed that indomethacin did not inherently augment the IL-2 response in positive control cultures. Furthermore, indomethacin did not inherently increase the proliferation of CTLL over background levels (data not shown).

### TBM and Prostaglandins

$PGE_2$ is made by a membrane-bound prostaglandin synthetase. We reasoned that antibody specific for

Depending on the stage of maturation, state of activation, and source, macrophages have been reported to either enhance or suppress immune responses (Stenson and Parker, 1980). Classically, macrophages are believed to be important accessory cells for antigen presentation (Rosenthal and Shevach, 1973a, 1973b; Unanue, 1981). A requirement for macrophages to function as accessory cells is the expression of MHC products. In contrast, in many cases, it has been shown that macrophages do not function as accessory cells but instead suppress immune reactions (Parkhouse and Dutton, 1966). In a series of publications by Lee and colleagues, it was suggested that the functional heterogeneity expressed by macrophages in the modulation of immune responses can be differentiated according to macrophage size as measured by velocity sedimentation rates (Lee and Berry, 1977; Lee and Wong, 1982; Guidos et al., 1987). Antigen-presenting activity was found to be characteristic of small macrophages, whereas large macrophages tended to be immunosuppressive. Furthermore, large macrophages that were induced to express class II molecules also exhibited suppressive effects on immune responses. Thus, the large size of TBM, even though they express class II (Smith et al., 1988) is compatible with the hypothesis that TBM may have a suppressive function to downregulate or limit the germinal center reaction.

Prostaglandins appear to be responsible for much of the immunosuppression mediated by macrophages. The suppressive effects of prostaglandins on immune responses are diverse and have been reviewed (Stenson and Parker, 1980; Goodwin and Ceuppens, 1983). The binding of immune complexes by macrophages stimulates phagocytosis, which in turn stimulates prostaglandin synthesis with the major product being $PGE_2$ with smaller amounts of $PGF_1$ (Bonney et al., 1979). This immune-complex stimulation may help explain the high level of prostaglandin in TBM since they may encounter immune complexes directly on the processes of FDC and in the form of iccosomes (Szakal et al., 1988). The data in this study show that indomethacin, an inhibitor of prostaglandin synthesis, abrogates the suppressive effect of the TBM preparation on B-cell antigen presentation. Controls indicated that indomethacin did not inherently augment the IL-2 response in the assay system (data not shown). A known suppressive effect of prostaglandins on immune responses is the inhibition of lymphokine synthesis by $T_H$ cells. Whereas most of the lymphocytes in germinal centers are B cells, a small number of T cells are present and most of these T cells are of the helper phenotype (Rouse et al., 1982a, 1982b). Interestingly, a close association between TBM and germinal center T cells has been observed (Nieuwenhuis and Opstelten, 1984, and unpublished).

Germinal centers are sites of memory B-cell production (Coico et al., 1983) and production of memory B cells involves somatic mutation and maintenance of B cells that express high-affinity receptors for the stimulating antigen (Wysocki et al., 1986). It has been suggested that B cells with low-affinity antigen receptors cannot compete for antigen persisting on FDC and are clonally deleted (Wysocki et al., 1986). The tingible bodies in TBM may represent phagocytized low-affinity B cells that have been condemned to undergo apoptosis (Manser et al., 1987; Schad and Phipps, 1988). Consistent with this hypothesis, Odartchenko et al. (1966) and Fliedner (1967) have reported that the majority of the tingible bodies located in TBM represent cells that have died either during late $G_2$ phase or just prior to a mitotic event.

It has also been suggested that TBM play an active role in initiating the germinal center reaction (Kamperdijk et al., 1978, 1982). This suggestion was based on the observations that TBM initially appeared at the onset of germinal center development and that peak numbers of TBM were seen at peak germinal center development. However, in recent studies, it has been shown that TBM are not absolutely essential for germinal center development. Germinal centers can develop in old mice in the absence of TBM (Smith et al., 1990). The data shown in this report are consistent with a regulatory role for TBM and it appears that TBM are more likely to down regulate than to stimulate the germinal center reaction.

### MATERIALS AND METHODS

### Animals

Ten- to sixteen-week-old female Balb/C (H-$2^d$) mice were purchased from Charles River Laboratories (Wilmington, MA). The animals were housed under pathogen-free conditions in standard cages equipped with filter tops and allowed food and water *ad libitum.*

### Cell Lines and mAb

The $T_{H1}$ cell hybridoma 3DO-54.8 was a gift from J. Kappler and P. Marrack (National Jewish Hospital, Denver). Cultures of 3DO-54.8 cells (3DO) produce IL-2 when stimulated by the antigen ovalbumin (OVA) recognized in the context of the MHC molecule I-$A^d$. The IL-2-dependent cytotoxic lymphocyte line CTLL used to assay IL-2 was provided by P. Allen and E. Unanue (Washington University, St. Louis). The B-cell hybridoma TA3 (I-$A^{k,d}$) was a generous gift from Laurie Glimscher. Supernatants from the hybridoma M3/38 (obtained from Hybritech, San Diego) was used as the source of anti-Mac-2 antibody. Anti-Thy-1 antibody was obtained from Pharmingen (San Diego). Recombinant human IL-2 was obtained from Southern Biotech.

### Immunizations

#### Active immunization.

Germinal center B cells were obtained from ovalbumin (OVA; #A5503, Sigma Chemical, St. Louis)-immune mice. Mice were injected with 0.5 mg of OVA emulsified with complete Freunds adjuvant (CFA, Difco Laboratories, Detroit) behind the neck followed by a second 0.5-mg injection of OVA in CFA 14 days later. Seven days prior to germinal center B-cell isolation, mice received challenge injections of 0.5 $\mu g$ OVA in all footpads. Anti-OVA sera was prepared in rabbits immunized subcutaneously with 1.0 mg OVA emulsified in CFA followed by a booster immunization without CFA. Rabbits were bled via ear veins 7 days after booster immunization.

#### Passive immunization.

Phagocytosis of Freunds adjuvant by TBM and other macrophages can affect their densities and can cause erroneous isolation and the identification of non-TBM macrophages as TBM. Therefore, to obtain a population of TBM not contaminated by Freunds-adjuvant-containing macrophages, mice were passively immunized by intraperitoneal injections of 0.5 ml of rabbit anti-OVA. Eighteen to twenty-four hours later, challenge injections of 8-10 $\mu g$ of OVA diluted in 0.9% phosphate-buffered saline (PBS), pH 7.4, were injected into all four footpads. By using this immunization protocol, germinal center development and TBM were first evident 3 days after the antigenic challenge. Optimal numbers of TBM were present 8-10 days after challenge (Smith et al., 1990; Tew et al., 1990).

### Isolation and Enrichment of TBM and B Cells

Techniques for obtaining enriched populations of TBM (Smith et al., 1988) and germinal center B cells (Kosco et al., 1988) were previously described in detail. Briefly, 8 to 10 days after antigen challenge of OVA immune mice, lymph nodes were collected, enzymatically dissociated, and enriched on a discontinuous Percoll (Pharmacia, Piscataway, NJ) gradient (densities 1.042 and 1.064). Cells at the 1.064 interface were collected, washed, and incubated in tissue-culture dishes (Costar #3100, Cambridge, MA) at 37°C for 45 min to remove strongly adherent cells, which were primarily typical macrophages. Weakly adherent cells, including TBM, were then flushed off the culture dishes using a Pasteur pipette and counted. This population consisted of 7-10% TBM, 50-60% B cells (including about 20% $PNA^{hi}$ GC B cells) and 30-35% T cells.

Enriched populations of germinal center B cells were obtained as previously described (Kosco et al., 1988). Briefly, lymph nodes from actively immunized mice were collected at 3 and 5 days after challenge with OVA, enzymatically dissociated, and enriched on a continuous Percoll gradient. Further enrichment was achieved by panning for germinal center B cells on peanut agglutinin (PNA; Sigma)-coated tissueculture dishes (Costar #3100). Unbound cells were removed by gentle swirling and PNA-positive germinal center B cells were then eluted from the dishes by adding 0.2 M d-galactose (Sigma) for 1 hr before vigorous flushing with a pipette. By using this technique, 70-80% enriched populations of highly $PNA^+$ germinal center B cells were commonly attained.

### Depletion of TBM Using Dynabeads

It is known that TBM are the only Mac-2-bearing cells in lymph nodes (Flotte et al., 1983; Smith et al., 1990), and, in addition, TBM also express Thy-1 (Smith et al., 1988). Consequently, TBM can be selectively removed from TBM-enriched cell populations using anti-Mac-2 and anti-Thy-1 on immunomagnetic beads (Dynal, Great Neck, NY). The beads were purchased precoated with Fc-specific sheep anti-rat IgG. The beads were washed five times, incubated with a cocktail of anti-Mac-2 (1:4 dilution) and mouse anti-rat Thy-1 (1:20) for 1 hr at 4°C with intermittent agitation, and gently washed three times with HBSS. The beads were then incubated with the Mac-2/Thy-1-treated TBM-enriched preparation at a bead-to-cell ratio of 40:1 for 30 min on ice with gentle intermittent agitation. Following incubation, the Dynabead rosetted TBM were removed using the Dynal magnetic chamber. The remaining cells were collected with a Pasteur pipette, washed, and adjusted to $2 \times 10^5$ cells/ 50 $\mu$l in complete media. This preparation was used as the TBM-depleted population.

### IL-2 Assay for Antigen Presentation

Antigen presentation was assessed using an assay system similar to the one originally described by Shimonkevitz et al. (1983). In this system, the OVA-specific $T_H$-cell hybridoma 3DO-54.8 (3DO) will produce the lymphokine IL-2 when cocultured with OVA and a source of antigen-presenting cells. The production of IL-2 can then be quantitated by transferring aliquots of media from the cocultured cells to the IL-2-dependent T-cell line CTLL. The presence of IL-2 in the media stimulates the proliferation of CTLL cells, which can be measured by $^3H$-thymidine incorporation. Ultimately, then, the proliferation of CTLL serves as an index for antigen presentation. From $5 \times 10^4$ to $1 \times 10^6$ antigen-presenting cells (germinal center B cells or TA3 cells) were cocultured with $3.0 \times 10^4$ 3DO cells in 96-well flat-bottom plates (Falcon #3596) at 37°C, in 5% $CO_2$ atmosphere for 24 hr in the presence of 50 $\mu$g of OVA. RPMI 1640 (GIBCO, Grand Island, NY) supplemented with 10% pyrogen-free fetal calf serum, 100 $\mu$g/ml gentamicin (GIBCO), 0.2 mM glutamine (GIBCO), and 10 mM Hepes buffer (GIBCO) was used as the culture media. Positive controls for antigen presentation were either (1) cocultures of $5 \times 10^5$ to $1.0 \times 10^6$ germinal center B cells with $5.0 \times 10^4$ 3DO cells in the presence of 50 $\mu$g of OVA or (2) $5.0 \times 10^4$ TA3 cells with $3.0 \times 10^4$ 3DO cells in the presence of 50 $\mu$g OVA.

To assess the production of IL-2 by 3DO cells, 150-$\mu$l aliquots of coculture media were transferred to $3 \times 10^4$ CTLL cells in 96-well U-bottom plates (Costar #3799) and cultured for 24 hr at 37°C, at 5% $CO_2$ atmosphere. At 16-20 hr, each well was pulsed with 1.0 $\mu$Ci $^3H$-thymidine (New England Nuclear, Boston) and harvested (PHD Harvester, Cambridge Technical) at 24 hr onto glass filter strips (Costar, #240-1). Proliferation of CTLL cells induced by IL-2 in the coculture media was then measured as thymidine incorporation using a Packard 2200CA scintillation counter. All culturing were performed in triplicate and the data were expressed as the mean $^3H$-thymidine uptake. Cultures of CTLL cells alone and supernatant from 3DO cells alone in the presence or absence of OVA were used as controls and were consistently negative (mean cpm ± standard error = 461 ± 76). As a control for T-cell contamination in the germinal center B-cell preparation, germinal center B cells were cultured alone in the presence of antigen. The germinal center B-cell preparation appeared to contain some T cells, as indicated by slight but inconsequential IL-2 production when cultured in the presence of antigen.

### Influence of TBM on Antigen Presentation by Germinal Center B Cells

To determine if TBM have an affect on the capacity of B cells to present antigen, 1-2 × $10^5$ TBM-enriched cells (containing 14,000-16,000 TBM) were cocultured with B cells (either 5.0 × $10^5$ to 1.0 × $10^6$ germinal center B cells or 5.0 × $10^4$ TA3 cells) and 3.0 × $10^4$ 3DO cells in the presence of 50 μg OVA. As in the antigen-presenting assay, in some cultures, TBM were removed from the enriched preparations using Mac-2-coated Dynabeads prior to culturing. In some experiments, 50 μl of 100 μM indomethacin (Sigma, St. Louis), a prostaglandin inhibitor, was added. Prostaglandins are known to inhibit IL-2 synthesis. As a control, to ensure that indomethacin did not inherently affect the assay, indomethacin was also added to cocultures of germinal center B cells, antigen, and 3DO cells in the absence of TBM-enriched cells.

### Immunocytochemistry

Reactivity for prostaglandins was detected on enriched preparations of TBM using indirect peroxidase immunocytochemistry. Paraformaldehyde/glutaraldehyde-fixed cytobucket (IEC) slide preparations of TBM-enriched populations were incubated for 30 min at 4°C with goat anti-human $PGE_2$ (1:30) (ICN, Irvine, CA) as the primary antibody. The slides were washed 3 × 5 min with 0.1 M cacodylate buffer followed by incubation with a peroxidase-conjugated rabbit anti-goat IgG (1:100) as the secondary antibody. Specific peroxidase activity was localized, using DAB as chromogen, and endogenous perox-

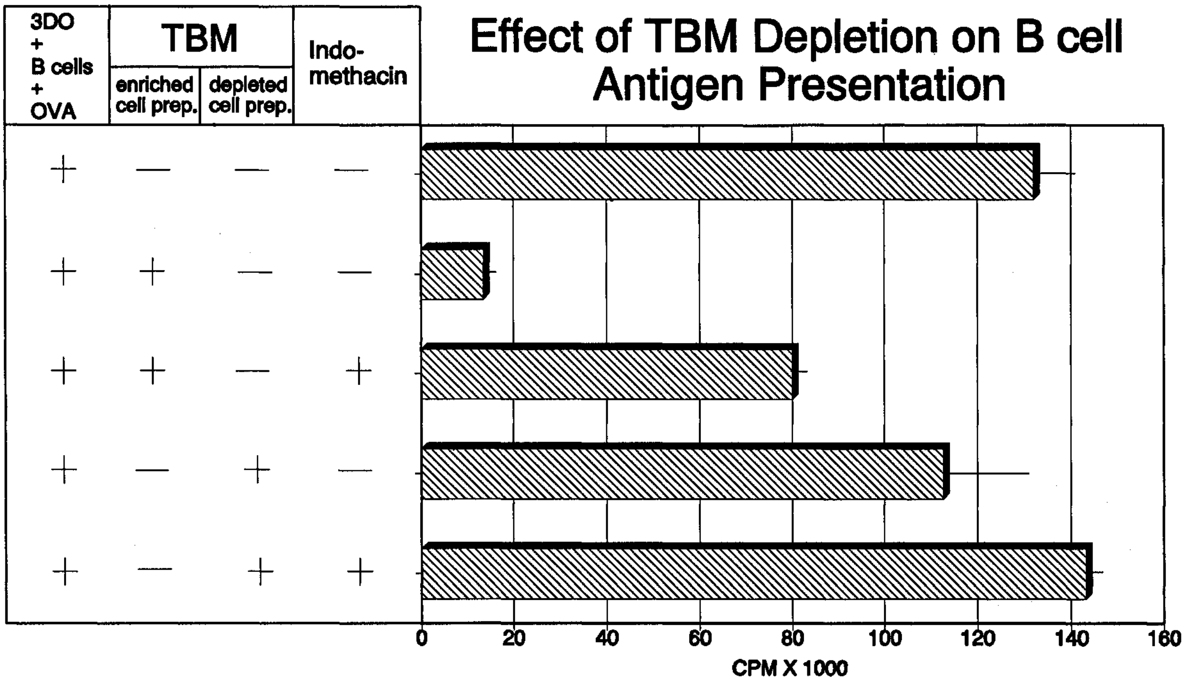

FIGURE 2 Effect of depletion of TBM from TBM-enriched populations on lymphokine secretion. TBM-enriched cells (1 × $10^5$), depleted of TBM by MAC-2/THY-1-coated Dynabeads (−TBM), or nondepleted (+TBM) were cocultured with positive antigen-presenting cultures (3DOs + B cells + OVA) in the presence or absence of 100 μM of indomethacin. Data represent proliferation of CTLL cells in response to IL-2 produced in coculture supernatants and are expressed as mean cpm ± standard error of triplicate cultures.

idase activity was inhibited according to the method of Herzog and Miller (1972).

### Depletion of TBM Relieves Suppression of IL-2 Activity

If the TBM were responsible for depressing the IL-2 production, depletion of the TBM from the enriched preparation prior to addition to the control cultures should alleviate the suppression. Results from a representative experiment using TBM-depleted cells are shown in Fig. 2. Addition of the TBM-enriched population to positive controls resulted in dramatic depression of IL-2 production—about 10% of positive control cultures. Addition of indomethacin to cultures containing TBM-enriched cells resulted in a restoration of most of the IL-2 activity. Furthermore, depletion of TBM from TBM-enriched populations restored IL-2 production to a level insignificantly less than that of the positive controls.

### *Acknowledgements*

This work was supported by National Institutes of Health grants AG 05374 and AI 17142.

### *References*

Bonney R.J., Narans P., Davies P. and Humes J.L. (1979). Antigen-antibody complexes stimulate the synthesis and releases of prostaglandins by mouse peritoneal macrophages. Prostaglandins **18**:605-616.

Coico R.F., Bhogal B.S. and Thorbecke G.J. (1983). Relationship of germinal centers in lymphoid tissue to immunologic memory. VI. Transfer of B cell memory with lymph node cells fractionated according to their receptors for peanut agglutinin. J. Immunol. **131**:2254-2257.

Flemming W. (1885). Studien uber regeneration de gewbe. Arch. Mikr. Anat. **24**:50.

Fliedner T.M. (1967). On the origin of tingible bodies in germinal centers. In Germinal Centers in Immune Responses, Cottier H., Odartchenko D., Schindler R. and Congdon C.C., eds. (New York: Springer-Verlag), pp. 218-221.

Flotte T.J., Springer T.A. and Thorbecke G.J. (1983). Dendritic cell and macrophage staining by monoclonal antibodies in tissue sections and epidermal sheets. Amer. J. Pathol. **111**:112-124.

Gillis S. and Smith K.A. (1977). Long term culture of tumor-specific cytotoxic T cells. *Nature* **268**:154-156.

Goodwin J.S. and Ceuppens J. (1983). Regulation of immune response by prostaglandins. *J. Clin. Immunol.* **3**:295-315.

Guidos C., Sinha A. and Lee K.-C. (1987). Functional differences and complementation between dendritic cells and macrophages in T cell activation. Immunology **61**:269-276.

Herzog V. and Miller F. (1972). Endogenous peroxidase in the lacrimal gland of the rat and its differentiation against injected catalase and horseradish peroxidase. Histochemie **30**:235-246.

Kamperdijk E.W.A., Raaymakers E.M., de Leeuw J.H.S. and Hoefsmit E.C.M. (1978). Lymph node macrophages and reticulum cells in the immune response. 1. The primary response to paratyphoid vaccine. Cell Tiss. Res. **192**:1-23.

Kamperdijk E.W.A., de Leeuw J.H.S. and Hoefsmit E.C.M. (1982). Lymph node macrophages and reticulum cells in the immune response. The secondary response to paratyphoid vaccine. Cell Tiss. Res. **227**:277-290.

Kosco M.H., Szakal A.K. and Tew J.G. (1988). *In vivo* antigen presented by germinal center B cells to T cells in vitro. J. Immunol. **140**:354-360.

Lee K.-C. and Berry D. (1977). Functional heterogeneity of macrophages activated by Corynebacterium parvum: Characterization of macrophages with different activities in promoting immune responses and suppressing tumor cell growth. J. Immunol. **118**:1530-1540.

Lee K.-C. and Wong M. (1982). Functional heterogeneity of culture-grown bone marrow-derived macrophages. II. Lymphokine stimulation of antigen-presenting function. J. Immunol. **128**:2487-2492.

Manser T., Parhami-Seren B., Margolies M.N. and Gefter M.L. (1987). Somatically mutated forms of a major anti-p-azophenylarsonate antibody variable region with drastically reduced affinity. J. Exp. Med. **166**:1456-1463.

Nieuwenhius P. and Opstelten D. (1984). Functional anatomy of germinal centers. Amer. J. Anat. **170**:421-435.

Odartchenko N., Lewerenz M., Sordat B., Roos B. and Cottier H. (1966). Kinetics of cellular death in germinal centers of mouse spleen. In Germinal Centers in Immune Responses, Cottier H., Odartchenko D., Schindler R. and Congdon C.C., eds. (New York: Springer-Verlag), pp. 212-217.

Parkhouse R.M.E. and Dutton R.W. (1966). Inhibition of spleen cell DNA synthesis by autologous macrophages. J. Immunol. **97**:663-669.

Rosenthal A.S. and Shevach E.M. (1973a). Function of macrophages in antigen recognition by guinea pig T lymphocytes. I. Requirement for histocompatible macrophages and lymphocytes. J. Exp. Med. **138**:1194-1212.

Rosenthal A.S. and Shevach E.M. (1973b). Function of macrophages in antigen recognition by guinea pig T lymphocytes. II. Role of the macrophage in the regulation of genetic control of the immune response. J. Exp. Med. **138**:1213-1218.

Rouse R.V., Ledbetter J.A. and Weissman I.L. (1982a). Mouse lymph node germinal centers contain a selected subset of T cells—The helper phenotype. J. Immunol. **128**:2243-2246.

Rouse R.V., Weissman I.L., Ledbetter J.A. and Warnke R.A. (1982b). Expression of T cell antigens by cells in mouse and human primary and secondary follicles. Adv. Exp. Med. Biol. **149**:751-756.

Schad V.C. and Phipps R.P. (1988). Two signals are required for accessory cells to induce B cell unresponsiveness: Toleragenic Ig and prostaglandin. J. Immunol. **141**:79-84.

Shimonkevitz R., Kappler J., Marrack P. and Grey H. (1983). Antigen recognition by H-2 restricted T cells. I. Cell free antigen processing. J. Exp. Med. **158**:303-316.

Smith J.P., Kosco M.H., Tew J.G. and Szakal A.K. (1988). Thy-1 positive tingible body macrophages (TBM) in mouse lymph nodes. Anat. Record **222**:380-390.

Smith J.P., Lister A.L., Tew J.G. and Szakal A.K. (1990). Kinetics of the tingible body macrophage response in mouse germinal center development and its depression with age. Anat. Record **229**:551-520.

Stenson W.F. and Parker C.W. (1980). Prostaglandins, macrophages and immunity. J. Immunol. **125**:1-5.

Swartzendruber D.C. and Congdon C.C. (1963). Electron microscopic observations on tingible body macrophages in mouse spleen. J. Cell Biol. **19**:641-646.

Szakal A.K., Kosco M.H. and Tew J.G. (1988). A novel *in vivo* follicular dendritic-cell dependent iccosome-mediated mechanism for delivery of antigen to antigen-processing cells. J. Immunol. **140**:341-353.

Szakal A.K., Kosco M.H. and Tew J.G. (1989). Microanatomy of lymphoid tissue during humoral immune responses: Structure function relationships. Annu. Rev. Immunol. **7**:91-109.

Tew J.G., Kosco M.H., Burton G.F. and Szakal A.K. (1990). Follicular dendritic cells as accessory cells. Immunol. Rev. **117**:185-211.

Unanue E.R. (1981). The regulatory role of macrophages in antigenic stimulation. Part Two: Symbiotic relationship between lymphocytes and macrophages. Adv. Immunol. **31**:1-136.

Wysocki L., Manser T. and Gefter M.L. (1986). Somatic evolution of variable region structures during an immune response. Immunology **83**:1847-1851.

